# Geospatial Distribution of Family Planning Services in Kira Municipality, Wakiso District, Uganda

**DOI:** 10.3389/fgwh.2020.599774

**Published:** 2021-02-10

**Authors:** Moses Tetui, Tonny Ssekamatte, Pierre Akilimali, Judith Sirike, Osvaldo Fonseca-Rodríguez, Lynn Atuyambe, Fredrick Edward Makumbi

**Affiliations:** ^1^School of Pharmacy, Waterloo University, Waterloo, ON, Canada; ^2^Department of Epidemiology and Global Health, Umeå University, Umeå, Sweden; ^3^Department of Health Policy, Planning and Management, Makerere University School of Public Health, New Mulago Hospital Complex, Kampala, Uganda; ^4^Department of Disease Control and Environmental Health, Makerere University School of Public Health, New Mulago Hospital Complex, Kampala, Uganda; ^5^Department of Nutrition Kinshasa School of Public Health, Faculty of Medicine, University of Kinshasa, Kinshasa, Democratic Republic of Congo; ^6^Division of Social Development, Intergovernmental Authority on Development, Kampala, Uganda; ^7^Centre for Demographic and Ageing Research, Umeå University, Umeå, Sweden; ^8^Department of Community Health and Behavioural Sciences, Makerere University School of Public Health, New Mulago Hospital Complex, Kampala, Uganda; ^9^Department of Epidemiology and Biostatistics, Makerere University School of Public Health, New Mulago Hospital Complex, Kampala, Uganda

**Keywords:** urban poor, family planning, informal settlements, Kira Municipality, Uganda

## Abstract

**Introduction:** Access to family planning (FP) services remains a challenge, particularly in informal urban settlements. The unmet need for FP in these settings is high, with a correspondingly high prevalence of unintended pregnancies that may lead to unsafe abortions. However, there is a paucity of quality data on the distribution of FP services in such settings in Uganda. This paper described the geospatial distribution of FP services in Kira Municipality, Wakiso District, Uganda.

**Methods:** This was a cross-sectional study in which we determined the availability and distribution of FP services in Kira Municipality. Community mapping and analysis were conducted using ArcGIS (version 10.1) and ArcGIS Online. Stata version 13.1 was used for data analysis. Chi-square test was used to compare the contraceptive provision and availability among facilities from informal and formal settlements.

**Results:** Of the 176 healthcare facilities surveyed, only 42% (*n* = 74) offered contraceptives in informal settlements. The majority of the facilities were privately owned small clinics (95%). At least 80% of the facilities provided three or more modern contraceptive methods, with no difference (*p* = 0.107) between facilities in informal and formal settlements. Only 30.7% (*p* = 0.001) of the facilities provided at least one long-acting contraceptive. Similarly, 20 and 12% (*p* = 0.001) of the facilities had implants and intrauterine devices (IUDs) on the day of the survey. Almost 25% of the facilities did not offer contraceptive services (counseling and commodities) to unmarried adolescents.

**Conclusions:** Most facilities were small privately-owned clinics, offering at least three modern contraceptive methods. The unavailability of long-acting reversible methods in the informal settings may affect the quality of FP services due to limited choice. The inequity in service provision that disfavors the unmarried adolescent may increase unwanted/unintended pregnancies. We recommend that local governments and partners work toward filling the existing commodities gap and addressing the discrimination against unmarried adolescents in such settings.

## Introduction

Family planning (FP) entails a conscious effort by a couple to limit or space the number of children they have through the use of contraceptive methods. The use of contraceptives is known to lower both maternal and infant mortality risks (1–6). The use of contraceptives also has the potential to reduce disabilities related to complications of pregnancy and childbirth (7). On the other hand, failure to meet the contraceptive needs often results in unintended pregnancies (2, 8). Nonetheless, globally, access to FP services remains a public health challenge. In 2018, it was reported that over 214 million women of reproductive age in developing countries still lacked access to a modern contraceptive method (9).

In 2015, <75 and <50% of the total demand for modern contraceptives in 76 countries and 54 countries (34 of which were in Africa), respectively, were met (10). The unmet need for FP remains the highest in low-income countries (5), with 22% of sexually active women not using a modern contraceptive method even when they desire to (10). Uganda is no exception in terms of the disparities in accessing FP services. Over 28% of currently married women and 32% of sexually active unmarried women have an unmet need for FP (11, 12). This figure falls below the country's commitment to reduce the unmet need for FP to below 10% by 2020 (12, 13). In addition, at least 48% of those who demand for FP in Uganda are not satisfied with modern contraceptive methods, whereas 43% stop using FP methods within 12 months of starting, mainly due to health concerns or fear of side effects and desire to return of fertility (12).

Country-level estimates also indicate that 52% of pregnancies in Uganda are unwanted or mistimed. Over 43% of unintended pregnancies are attributable to the unmet need for FP (11, 12). Unintended pregnancies often result in unplanned births, unsafe abortions, and maternal injury and death (14–17). Despite the negative consequences of unplanned pregnancies (18), women who want to avoid pregnancy still have an unmet need for FP (13).

The limited utilization of modern contraceptives stems from religious and socio-cultural barriers such as uncooperative spouses; perceptions of poor quality of services, limited choice and access to contraceptives (5, 19, 20), users and providers perceptions, and gender-based barriers (9, 21) and fear or experience of side effects (17, 22). Such constraints affect vulnerable populations disproportionately. People living in informal settlement settings are often poor and more vulnerable to adverse health events compared with their other urban counterparts (23).

Increasing urbanization creates a heavy toll on the urban social services that are already severely constrained, owing to a less than proportionate growth of the needed urban planning for the increasing number of residents (23). For example, public health facilities have not increased in number in most urban spaces despite the increasing urbanization. This has created a gap that the private sector has exploited to provide the needed services, including modern contraceptives. With a very mixed health care system that has a weakly regulated private sector in the lead, urban residents often suffer stark quality issues, which often affect the urban poor more severely (24). Ironically, people living in urban places are always assumed to have better access to services and information; hence, interventions targeting urban places are often fewer (25). They are often caught in a series of trial care-seeking practices, which makes them even more vulnerable financially in what (26) called “Money for nothing” in their paper on dire medical practices, especially among the private practitioners. Additionally, existing evidence indicates that the urban poor are faced with even more complex issues related to health care services, including sanitation, reproductive health services in general, and FP specifically (27, 28).

Failure to implement impactful FP programs has led to an increase in infertility rates with its associated outcomes in informal settings (29, 30). An increase in fertility rates is also associated with a reduction in household income (29). To put this into context, over 70% of all urban residents in the Sub-Saharan Africa (SSA) live in informal settlements. These settlements are characterized by extreme poverty and have poor economic, maternal, and child health indicators (29). Mberu et al. (29) point out that child residents in these areas are at a higher risk of suffering from childhood illnesses and malnutrition and bear a disproportionately much higher mortality burden than their counterparts in other urban and rural settings. Within the urban poor, adolescents face unique challenges with regard to access to FP services. Societal norms and beliefs, for instance, limit their access and utilization of FP services, thereby leaving them at an elevated risk of teenage pregnancy and sexually transmitted infections (31).

In addition, existing literature indicates that the cost of transport, misinformation and misconceptions, religious opposition, and provider biases further inhibit access to FP services in informal settlements. However, there is still limited evidence on the availability and distribution of FP services within informal settlements. Yet, geographical information systems (GISs) can be used to improve health service programming in such challenging environments (32, 33). This study, therefore, sought to illustrate the geospatial distribution of FP providers, as well as the different FP services, in Kira Municipality with a special focus on informal settlements. These findings can be used to inform programs that aim at scaling up access and utilization of FP services.

## Materials and Methods

### Study Setting

**Figure 1:**
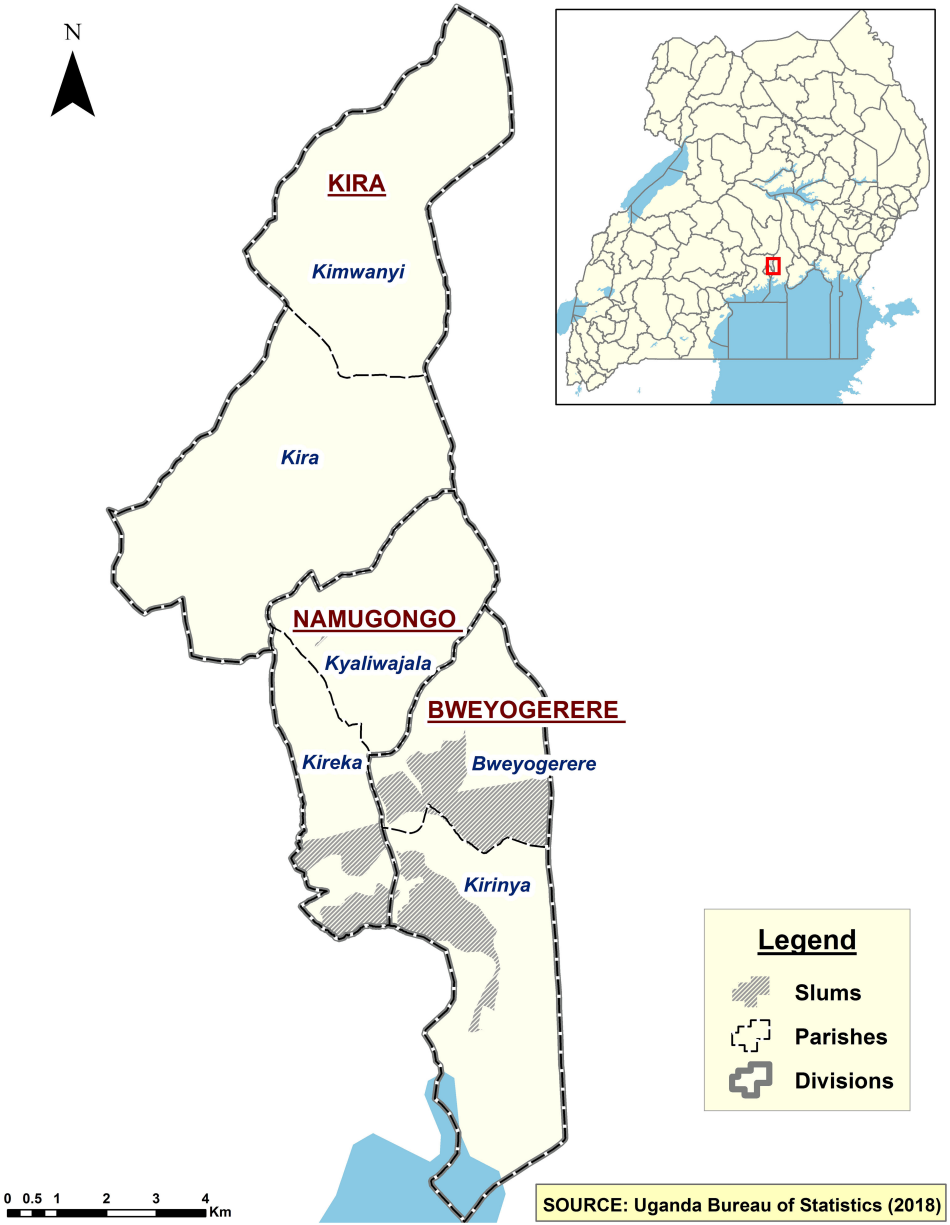
Map of Kira Municipality indicating its geographical demarcations.

The study was undertaken in Kira Municipality in Wakiso District, Uganda. Wakiso District is composed of four municipal councils (Entebbe, Nansana, Kira, and Makindye Sabagabo), nine town councils (Kasanje, Kakiri, Kasangati, Kajjansi, Katabi, Kyengera, Masulita, Namayumba, and Wakiso), and six sub-counties (Bussi, Kakiri, Masulita, Namayumba, Mende, and Wakiso). The study was conducted in Kira Municipality, which was randomly selected from the four municipalities that form Wakiso District. Although the four municipalities are similar in some aspects, such as population, composition, population size, and social economic activities, just like all other urban settings, differences still exist across all social, economic, and political structures (34). Nonetheless, lessons drawn from Kira among the urban poor will still be relevant across other poor urban residents elsewhere. Kira Municipality has a growing population of over 400,000 residents (11). The municipality is divided into three divisions, Namugongo, Bweyogerere, and Kira Divisions. The Bweyogerere and Namugongo Divisions contain the informal settlements within Kira Municipality (Figure 1).

### Study Design and Data Collection

This was a cross-sectional study in which mapping and data collection was undertaken to locate the FP service points across the entire Kira Municipality. All FP service points in the municipality were targeted for the survey; all those whose caretakers consented (5/181 declined) were included in the study. Our study population therefore, was all FP service points at different levels of service, these included; general hospitals, health centers at levels IV, III, and II, pharmacies, clinics, and drug shops (35) and their caretakers. The data were collected by trained research assistants, who were familiar with Kira Municipality. In addition, local guides who worked at the municipality supported the research assistants to easily identify the facilities for the mapping exercise. KoboCollect, a mobile data collection application, was used to collect the data. Before data collection, the questionnaire was initially pre-tested around the Makerere University area, the area has both formal and informal settlements, which made it ideal, given its similarities with the actual study site. Following the pre-test, to ensure quality, the data entry screen was designed with skips and more restrictions added to ensure the completeness of entry. In addition, secondary mapping data (shapefiles for the administrative units) were obtained from the Uganda Bureau of Statistics spatial data portal (https://www.ubos.org/data-portals-2/).

The general aim of the maps was to show the spatial distribution of the health facilities in addition to producing information that could respond to specific questions, i.e., under what category of ownership are the health facilities that provide FP services?, and what is the level of facilities that provide FP services?

On a daily basis, the completed questionnaires were uploaded to a remote server to which only the principal investigator (PI) and study coordinator had access. The data were kept strictly confidential and later shared with an ArcGIS expert for analysis after removing all possible identifiers.

### Data Analysis

The data were downloaded into an Excel spreadsheet from the remote server. The Excel spreadsheet was then exported into a GIS environment as a comma-delimited (.csv) file and later into a vector file that was used to generate the required maps.

Data mapping and analysis were conducted using ArcGIS (version 10.1). Ground-truthing was carried out to relate the features on the ground with those on the satellite image in order to establish the location of informal settlements within Kira Municipality. This involved capturing geographic coordinates of sample spots within the municipality. These coordinates, as well as the shapefile, of Kira Municipality were later overlaid on the base map from ArcGIS that showed the area extent of Kira Municipality. Together with secondary data from the Uganda Bureau of Statistics and the ground-truth data, digitization was done within the GIS environment to create vector data that showed the extent of the informal settlements. Digital forms were designed to collect locational data as well as selected attribute datasets. After data collection, these data were exported to the GIS platform where further spatial analyses were executed using the overlay, buffer, and site selection tools, thus resulting in maps. Additional data on the specific services provided and availability on the day of the survey were analyzed in Stata for Macs version 13.1. Chi-square test was used to compare the contraceptive provision and availability among facilities from informal and formal settlements by assessing the (i) proportion of facilities providing modern contraceptives, (ii) availability of modern contraceptives in facilities, (iii) provision of at least one long-acting reversible modern contraceptives, (iv) provision of at least one short-acting modern contraceptive, (v) availability of intrauterine devices (IUDs) in facilities on the day of the survey, (vi) availability of the implant in facilities on the day of the survey, and (vii) proportion of facilities providing contraceptive services to unmarried adolescents.

## Results

### Distribution of Healthcare Facilities That Offer Modern Contraceptive Methods in Kira Municipality

**Figure 2:**
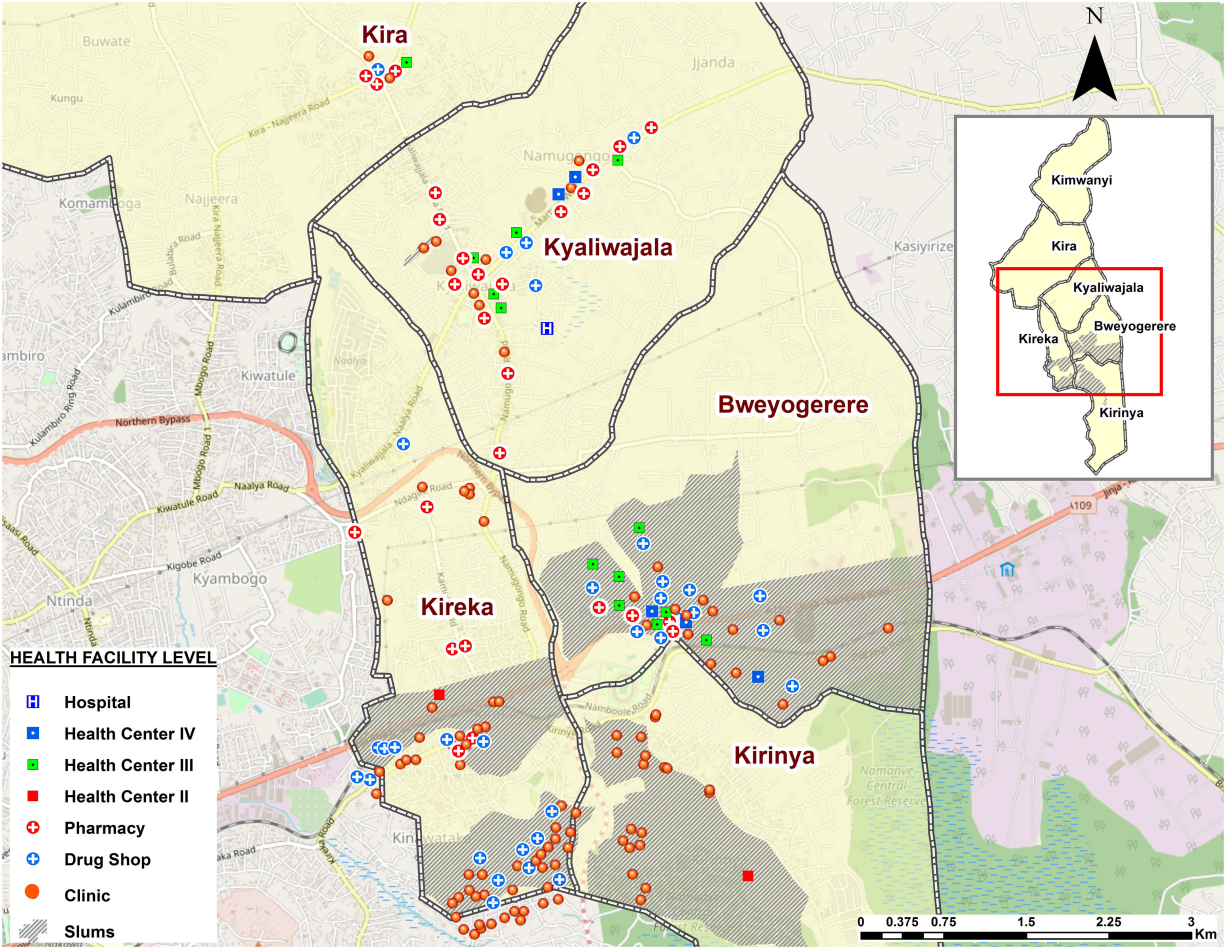
Map illustrating the type of health facilities found in Kira Municipality by level of care.

About 42% (74/176) of the facilities that offer contraceptives in Kira Municipality are found in informal settlements. In terms of the level of care of the facilities, the majority were small 1–3 roomed facilities or drug shops as indicated in Figure 2. Informal settlements had more of the 1–2 roomed (68.4%, 39/57) facilities, whereas formal settlements generally had more of the 3 roomed or bigger facilities (70.6%, 84/119). Over 94% (167/176) of the facilities were privately owned, of which 41.9% (70/167) were found in informal settlements. Additionally, 77% reported providing services on every day of the week, out of which 44.9% (79/176) were found in formal settlements, and only 32.4% (57/176) were found in informal settlements.

### Delivery of Family Planning Services

**Figure 3:**
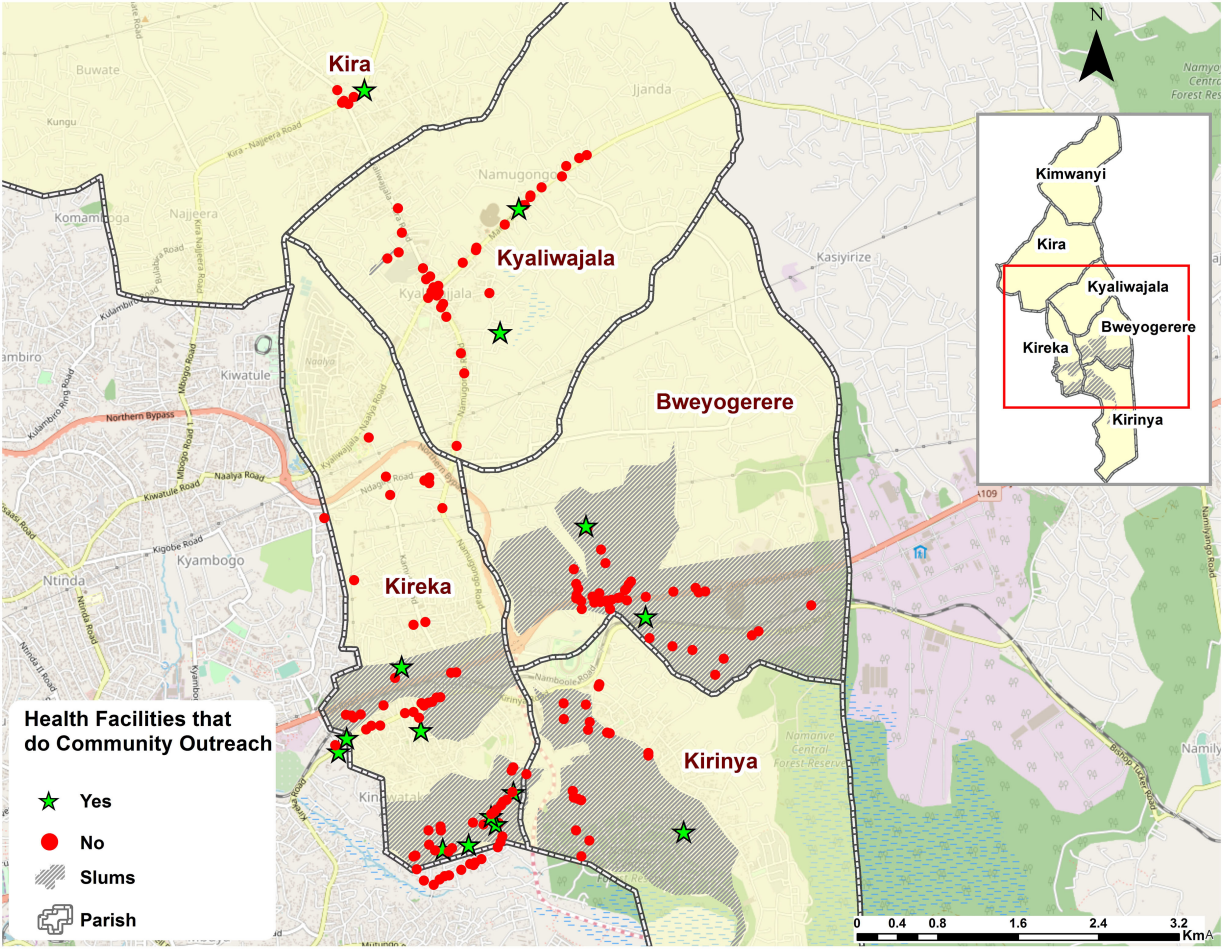
Map showing the facilities that provide out reaches for family planning services.

The facilities were geographically clustered, with clinics and drug shops in close proximity to each other and off the primary roads and deeper into informal settlements (Figure 3), whereas public health facilities (HC-III and -IV) were more in close proximity with pharmacies, especially in and along primary roads (Figure 2). In terms of mode of FP service delivery, over 90% (160/176) of the facilities did not provide outreach services as indicated in Figure 3. Of these, 43% (69/160) were found in informal settlements. Relatedly, more than 48% (86/176) of the facilities denied the researchers access to their examination rooms and premises for observation. Of the facilities that granted access for observations (51.1%, 90/176) to take place, <30% (25/90) of them had cues for action, such as information, education, and communication (IEC) materials related to FP displayed in their premises of operation. Of the 25 facilities, 56% (14/25) were found in informal settlements.

### Provision of Modern Contraceptives

The 81.3% of the facilities reported to providing three or more modern contraceptives. While there was no difference between the facilities in informal and formal settlements in terms of provision of contraceptives, there was a significant difference when it came to the provision of long-acting revisable contraceptives (implants and IUDs). Fewer facilities in informal settlements provided long-acting revisable methods (Table 1). Additionally, only three facilities provided permanent methods; all of them were found in formal settlements. The provision of short-acting modern contraceptives was similar in informal and formal settlements (see Table 1).

**Table 1:** Contraceptive provision and availability among facilities found in informal settlements compared with those in formal settlements.

**Variables**		**Informal settlements**	**Formal settlements**	**Overall**	**Chi2 (*p*-value)**
Proportion of facilities providing modern contraceptives	Less than three methods	18 (24.3%)	15 (14.7%)	33 (18.8%)	2.6044 (0.107)
	Three or more methods	56 (75.7%)	87 (85.3%)	143 (81.3%)	
Availability of modern contraceptives in facilities	Less than three methods	40 (54.1%)	46 (45.1%)	86 (48.9%)	1.3767 (0.241)
	Three or more methods	34 (45.9%)	56 (54.9%)	90 (51.1%)	
Provision of at least one long-acting reversible modern contraceptive	No	61 (82.4%)	61 (59.8%)	122 (69.3%)	**10.3235 (0.001)**
	Yes	13 (17.6%)	41 (40.2%)	54 (30.7%)	
Provision of at least one short-acting modern contraceptive	No	8 (10.8%)	7 (6.9%)	15 (8.5%)	0.8574 (0.354)
	Yes	66 (89.2%)	95 (93.1%)	161 (91.5%)	
Availability of the IUD in facilities on the day of the survey	No	20 (90.9%)	82 (54.0%)	102 (58.6%)	**10.8240 (0.001)**
	Yes	2 (9.1%)	70 (46.1%)	72 (41.4%)	
Availability of the implant in facilities on the day of the survey	No	29 (82.9%)	73 (52.5%)	102 (58.6%)	**10.6098 (0.001)**
	Yes	6 (17.1%)	66 (47.5%)	72 (41.4%)	
Proportion of facilities providing contraceptive services to unmarried adolescents	No	19 (25.7%)	24 (23.5%)	43 (24.4%)	0.1070 (0.744)
	Yes	55 (74.3%)	78 (76.5%)	133 (75.6%)	

*Statistically significant values are bold*.

### Provision of Modern Contraceptive Services to Unmarried Adolescent Women

In terms of the provision of services to unmarried adolescents, a significant number (24.4%) of facilities did not offer contraceptive services to this group of people. The facilities did not offer counseling about nor the modern contraceptives to these unmarried adolescents. While the majority (75.6%) provided the service to unmarried adolescents, a significant number did not. No significant difference was found between the facilities in the different settlement areas regarding the provision of modern contraceptive services to unmarried adolescents (Table 1).

### Availability of Modern Contraceptives

On the day of the survey, nearly half (48.9%, 86/176) of the facilities had less than three methods in stock as reported by the respondents. There was no significant difference noted between the areas of settlement when all the methods were put into consideration. However, a look at the availability of long-acting revisable methods indicated that fewer facilities in the informal settlements had both the implant and IUD available on the day of the survey (Table 1).

## Discussion

This section reflects on the distribution of facilities that provide FP service in and outside informal settlement settings, FP provision infrastructure, mode of delivery of FP services, and types of FP services provided and available on the day of the survey. This study indicates that informal settlements are mainly serviced by drug shops and small clinics. This may not be surprising given the insecure land tenure system that characterizes most informal settlements. Insecure land tenure systems in informal settlements could deter the government and the greater private sector players from investing in the construction of bigger infrastructure, such as hospitals or bigger health centers (36). Besides insecure land tenure systems, investments in such areas may not be as profitable mainly due to the lower socio-economic status of the residents, thereby limiting prospective investors (37–40).

Conversely, much bigger healthcare facilities if constructed in informal settlements would require a large number of staff and higher remuneration. Therefore, due to these operational challenges, service providers often resort to renting small rooms that they use as clinics, or drug shops rather than establishing permanent infrastructure to serve as higher level healthcare facilities, particularly hospitals and health center IVs. Lower-level healthcare facilities can easily enable them to break-even compared with higher level and bigger healthcare facilities. Failure to establish high level healthcare facilities, such as hospitals and health center IVs, has implications on contraceptive choice and the unmet need for FP. For instance, permanent FP methods, such as tubal ligation, vasectomy, and the long-acting revisable methods (IUD and implants), may only be provided in a healthcare facility with an operating room/theater and appropriately trained medical personnel. This is in contrast with the *status quo* in the drug shops and clinics that are widely spread in informal settlements and largely lacking these facilities and personnel.

In this study, majority of healthcare facilities that offer FP services are privately owned. Privately owned healthcare facilities often aim at maximizing profits implying that people residing in these informal settlements have to pay to access contraceptive services, hence limiting accessibility. The majority of the healthcare facilities in informal settlements only offer static FP services, a model that limits access to those who do not have sufficient time to access healthcare facilities, particularly for long-acting contraceptive methods. Having the possibility of offering outreach services could be inhibited by resource constraints given the nature of providers in informal settlements. Outreach services could theoretically increase access to knowledge on the use of contraceptive services given the highly mobile nature of informal settlement residents and the infrastructurally inaccessible locations of the settlements (41, 42).

Dissemination of information regarding the use of the different contraceptive methods remains poor in most low- and middle-income settings. Informal settlements in Kira Municipality are no exception, almost a third of the surveyed healthcare facilities did not display any IEC materials related to FP displayed in their premises of operation. Displaying IEC materials on FP is often an indicator to the clients that a facility offers a particular service (43). In addition, displaying such materials can boost the confidence of the client seeking a contraceptive and may act as a catalyst for further engagement. Having IEC materials displayed in a healthcare facility may also reduce stigma among clients, particularly among adolescents and young people who are often scared of asking for particular FP services. Healthcare facilities that lack such materials are often shunned by adolescents and young people, as well as those who cannot use native languages, such as refugees.

This study reaffirms that contraceptive choice among urban residents is still a big challenge, and more so among people living in informal settlements. Majority of the healthcare facilities in informal settlements offer more than three contraceptive methods. However, the contraceptive method mix was limited, which is in agreement with a finding of Ochako et al., (44), which indicated that the contraceptive choices of women living in Kenyan informal settings were constrained. Limited access to a wide range of contraceptive methods, particularly long-acting revisable and permanent methods, implies that residents of informal settings often have constrained access to contraceptive choices, which could discourage use (45).

Additionally, this limitation to contraceptive choice for women living in formal settings implies that they often have to use short-acting contraceptives, these are known to offer limited protection and increased chances of discontinuation by the users (44). The limited availability of long-acting revisable contraceptive methods in informal settlements could be attributed to their low demand and a low level of knowledge on these methods (46). Which further demonstrates disparities in access between the rich and the poor (47, 48). The use of long-acting revisable contraceptive methods has been found to be more efficacious compared to short-acting methods. An increase in their use is more likely to reduce costs associated with abortions, unwanted pregnancy, and unintended births. Ultimately, increased access to long-acting contraceptives will increase protection and reduce total fertility, therefore giving people living in informal settlements a chance to fight the vicious circle of poverty that is often exacerbated by large unplanned families (49).

Lastly, a considerable number (25%) of facilities indicated that they do not provide contraceptives to unmarried adolescents. Failure to provide contraceptives to adolescents worsens their vulnerabilities, a situation that is even worse among those living in informal settlements given their social-economic status (50, 51). Because of their comparatively poorer social-economic status, unmarried adolescents living in informal settlements will be more prone to sexual assault and would engage in more risky sexual behavior (52). These risks expose them to unwanted pregnancies and could consequently lead to unsafe abortions and ultimately poorer maternal and neonatal health outcomes, including death (50). Besides, failure to provide counseling services limits adolescents from making informed contraceptive choices and increases chances of increased fertility and its associated lowered standards of living.

### Study Limitations

The study did not examine the demand side aspects of contraceptive use in this setting, thereby making it difficult to assess the actual effect of the geographical distribution of FP services on modern contraceptive use.

## Conclusion and Recommendation

Small privately owned service delivery points (clinics), offering at least three modern contraceptive methods, were common in informal urban settings. However, the unavailability of long-acting reversible methods, the inequity in service provision that disfavors the unmarried adolescent, and the clustering of service delivery services may be clear indicators of poor FP quality services that affect choice and physical access. Such quality challenges can increase unwanted/unintended pregnancies in poor settings, especially among the unmarried sexually active adolescents.

Our findings are suggestive of the need for the different FP stakeholders (implementing partners and the ministry of health) to take into account the need to provide a wide range of contraceptives. Similarly, paying keen attention to the access disparities that mainly affect the urban poor and special groups, such as unmarried adolescents, will be imperative.

## Data Availability Statement

The raw data supporting the conclusions of this article will be made available by the authors, without undue reservation.

## Ethics Statement

The studies involving human participants were reviewed and approved by Makerere University School of Public Health Higher Degrees and Research Ethics Committee (HDREC), and the Uganda National Council of Science and Technology (UNCST). The patients/participants provided their written informed consent to participate in this study.

## Author Contributions

MT conceptualized the study. MT and TS participated in the data collection and drafted the manuscript. JS participated in the data collection, management, and analysis. PA and OF-R supported the data analysis process. LA contributed to the conceptualization of the study. FM provided overall technical guidance to the conceptualization process and participated in drafting the manuscript. All authors reviewed the manuscript, provided substantial input, and approved the final manuscript.

## Conflict of Interest

The authors declare that the research was conducted in the absence of any commercial or financial relationships that could be construed as a potential conflict of interest.

## References

[1] Ajong AB Njotang PN Yakum MN Essi MJ Essiben F Eko FE. Determinants of unmet need for family planning among women in Urban Cameroon: a cross sectional survey in the Biyem-Assi Health District, Yaoundé. BMC Women's Health. (2015) 16:4. 10.1186/s12905-016-0283-9 26791410 PMC4721192

[2] Tsui AO McDonald-Mosley R Burke AE. Family planning and the burden of unintended pregnancies. Epidemiol Rev. (2010) 32:152–74. 10.1093/epirev/mxq012 20570955 PMC3115338

[3] Jiang H Xu J Richards E Qian X Zhang W Hu L. Opportunities, challenges and systems requirements for developing post-abortion family planning services: perceptions of service stakeholders in China. PLoS ONE. (2017) 12:e0186555. 10.1371/journal.pone.0186555 29045434 PMC5646849

[4] Nuwasiima A Nuwamanya E Navvuga P Babigumira JU Asiimwe FT Lubinga SJ. Study protocol: incentives for increased access to comprehensive family planning for urban youth using a benefits card in Uganda. A quasi-experimental study. Reprod Health. (2017) 14:1–10. 10.1186/s12978-017-0400-8 29078815 PMC5659021

[5] Azmat SK Ali M Ishaque M Mustafa G Hameed W Khan OF. Assessing predictors of contraceptive use and demand for family planning services in underserved areas of Punjab province in Pakistan: results of a cross-sectional baseline survey. Reprod Health. (2015) 12:25. 10.1186/s12978-015-0016-9 25880987 PMC4383051

[6] Hazarika I. Women's reproductive health in slum populations in India: evidence from NFHS-3. J Urban Health. (2010) 87:264–77. 10.1007/s11524-009-9421-0 20148311 PMC2845837

[7] Institute G. Abortion and Postabortion Care in Uganda. (2017). Available online at: https://www.guttmacher.org/sites/default/files/factsheet/abortion-and-postabortion-care-uganda.pdf (accessed March 16, 2019).

[8] Bearak J Popinchalk A Alkema L Sedgh G. Global, regional, and subregional trends in unintended pregnancy and its outcomes from 1990 to 2014: estimates from a Bayesian hierarchical model. Lancet Glob Health. (2018) 6:e380–9. 10.1016/S2214-109X(18)30029-9 29519649 PMC6055480

[9] WHO. Family Planning/Contraception Fact Sheet. World Health Organisation (2018). Available online at: https://www.who.int/news-room/fact-sheets/detail/family-planning-contraception (accessed March 13, 2019).

[10] UN DESA. Trends in Contraceptive Use Worldwide 2015. UN, Department of Economic and Social Affairs, Population Division (2015). Available online at : http://www.un.org/en/development/desa/pop/publications/dataset/Contraception/wcu2015.shtml (accessed March 2, 2020).

[11] UBOS I. Uganda Demographic and Health Survey 2016: Key Indicators Report. Kampala (2017).

[12] Health MO. Investment Case for Reproductive, Maternal, Newborn, Child and Adolescent Health Sharpened Plan for Uganda 2016/17 – 2019/20 (2016).

[13] FP 2020. Uganda Commitment Maker Since 2012. Family Planning 2020 (2018). Available online at: http://www.familyplanning2020.org/uganda (November 23, 2018).

[14] Hussain R. Unintended pregnancy and abortion in Uganda. Issues Brief. (2013) 2:1–8. 23550324

[15] Beguy D Mumah J Gottschalk L. Unintended pregnancies among young women living in urban slums: evidence from a prospective study in Nairobi City, Kenya. PLoS ONE. (2014) 9:e101034. 10.1371/journal.pone.0101034 25080352 PMC4117474

[16] Sedgh G Bearak J Singh S Bankole A Popinchalk A Ganatra B. Abortion incidence between 1990 and 2014: global, regional, and subregional levels and trends. Lancet. (2016) 388:258–67. 10.1016/S0140-6736(16)30380-4 27179755 PMC5498988

[17] Mumah J Kabiru CW Izugbara CO Mukiira C. Coping with Unintended Pregnancies: Narratives from Adolescents in Nairobi's Slums. STEP UP Research Report. (2014) 10.31899/rh4.1053

[18] Beguy D Ezeh AC Mberu BU Emina JBO. Changes in use of family planning among the urban poor: evidence from Nairobi Slums. Popul Develop Rev. (2017) 43:216–34. 10.1111/padr.12038

[19] Tuloro T Deressa W Ali A Davey G. The role of men in contraceptive use and fertility preference in Hossana Town, Southern Ethiopia. Ethiopian J Health Develop. (2006) 20:152–9. 10.4314/ejhd.v20i3.46826

[20] Che Y Dusabe-Richards E Wu SC Jiang Y Dong XJ Li J. A qualitative exploration of perceptions and experiences of contraceptive use, abortion and post-abortion family planning services (PAFP) in three provinces in China. BMC Womens Health. (2017) 17:113. 10.1186/s12905-017-0458-z 29157259 PMC5697166

[21] Haffejee F O'Connor L Govender N Reddy P Sibiya MN Ghuman S. Factors associated with unintended pregnancy among women attending a public health facility in KwaZulu-Natal, South Africa. South Afr Family Pract. (2018) 60:79–83. 10.1080/20786190.2017.1396790

[22] Kabagenyi A Jennings L Reid A Nalwadda G Ntozi J Atuyambe L. Barriers to male involvement in contraceptive uptake and reproductive health services: a qualitative study of men and women's perceptions in two rural districts in Uganda. Reprod Health. (2014) 11:21. 10.1186/1742-4755-11-21 24597502 PMC3946591

[23] Satterthwaite D. The impact of urban development on risk in sub-Saharan Africa's cities with a focus on small and intermediate urban centres. Int J Disaster Risk Reduct. (2017) 26:16–23. 10.1016/j.ijdrr.2017.09.025

[24] Fotso JC. Child health inequities in developing countries: differences across urban and rural areas. Int J Equity Health. (2006) 5:9. 10.1186/1475-9276-5-9 16831231 PMC1544325

[25] Vlahov D Freudenberg N Proietti F Ompad D Quinn A Nandi V. Urban as a Determinant of Health. J Urban Health. (2007) 84:I16–26. 10.1007/s11524-007-9169-3 17356903 PMC1891649

[26] Das J Hammer J. Money for nothing: the dire straits of medical practice in Delhi, India. J. Dev. Econ. (2007) 83:1–36.

[27] Renzaho AM Kamara JK Georgeou N Kamanga G. Sexual, reproductive health needs, and rights of young people in slum areas of Kampala, Uganda: a Cross Sectional Study. PLoS ONE. (2017) 12:e0169721. 10.1371/journal.pone.0169721 28107371 PMC5249247

[28] Hebert LE Schwandt HM Boulay M Skinner J. Family planning providers' perspectives on family planning service delivery in Ibadan and Kaduna, Nigeria: a qualitative study. J Fam Plann Reprod Health Care. (2013) 39:29–35. 10.1136/jfprhc-2011-100244 22906857

[29] Mberu BU Haregu TN Kyobutungi C Ezeh AC. Health and health-related indicators in slum, rural, and urban communities: a comparative analysis. Glob Health Action. (2016) 9:33163. 10.3402/gha.v9.33163 27924741 PMC5141369

[30] Ishida K Stupp P Melian M. Fertility decline in Paraguay. Stud Fam Plann. (2009) 40:227–34. 10.1111/j.1728-4465.2009.00205.x 19852412

[31] Atuyambe LM Kibira SP Bukenya J Muhumuza C Apolot RR Mulogo E. Understanding sexual and reproductive health needs of adolescents: evidence from a formative evaluation in Wakiso district, Uganda. Reprod Health. (2015) 12:35. 10.1186/s12978-015-0026-7 25896066 PMC4416389

[32] Bertrand JT Kayembe P Dikamba N Mafuta E Hernandez J Hellen J. Using mapping of service delivery sites to increase contraceptive availability in Kinshasa, Democratic Republic of the Congo. Int Perspect Sex Reprod Health. (2014) 40:95–9. 10.1363/4009514 25051581

[33] Rosero-Bixby L. Spatial access to health care in Costa Rica and its equity: a GIS-based study. Soc Sci Med. (2004) 58:1271–84. 10.1016/S0277-953600322-8 14759675

[34] Satterthwaite D Owen DL. Outside the Large Cities: The Demographic Importance of Small Urban Centres and Large Villages in Africa, Asia and Latin America. London: IIED (2006).

[35] Tetui M Coe A-B Hurtig A-K Bennett S Kiwanuka SN George A. A participatory action research approach to strengthening health managers' capacity at district level in Eastern Uganda. Health Res Policy Syst. (2017) 15:110. 10.1186/s12961-017-0273-x 29297346 PMC5751402

[36] Richmond AK. Water, land, and governance: environmental security in dense Urban Areas in Sub-Saharan Africa. In: Francis G. Environment-Conflict Nexus. Villanova: Springer (2019). p. 91–102.

[37] Pryer JA. Poverty and Vulnerability in Dhaka Slums: The Urban Livelihoods Study. London: Routledge (2017).

[38] Brown AM. Uganda's new urban policy: participation, poverty, and sustainability. In: Proceedings of the Sustainable Futures: Architecture and Urbanism in the Global South. Kampala (2012). p. 27–30.

[39] Van Leeuwen JM Sekeramayi T Martell C Feinberg M Bowersox-Daly S. A baseline analysis of the Katanga Slums: informing urban public policy in Kampala, Uganda. Afr Popul Stud. (2017) 31:3845–54. 10.11564/31-2-1057

[40] Stephenson R Hennink M. Barriers to family planning service use among the urban poor in Pakistan. Asia Pacific Popul J. (2004) 19:5–26. 10.18356/e54a6ab6-en

[41] Al-Attar GST Bishai D El-Gibaly O. Cost-Effectiveness analysis of family planning services offered by mobile clinics versus static clinics in Assiut, Egypt. Afr J Rep Health. (2017) 21:30–8. 10.29063/ajrh2017/v21i1.2 29595023

[42] Jarvis L Wickstrom J Shannon C. Client perceptions of quality and choice at static, mobile outreach, and special family planning day services in 3 African countries. Glob Health Sci Pract. (2018) 6:439–55. 10.9745/Ghsp-D-18-00047 30287527 PMC6172111

[43] Puri MC Maharjan M Pearson E Pradhan E Dhungel Y Khadka A. Delivering postpartum family planning services in Nepal: are providers supportive? BMC Health Serv Res. (2018) 18:948. 10.1186/s12913-018-3777-3 30522481 PMC6282334

[44] Ochako R Izugbara C Okal J Askew I Temmerman M. Contraceptive method choice among women in slum and non-slum communities in Nairobi, Kenya. BMC Womens Health. (2016) 16:35. 10.1186/s12905-016-0314-6 27405374 PMC4941019

[45] Ezeh AC Kodzi I Emina J. Reaching the urban poor with family planning services. Stud Fam Plann. (2010) 41:109–16. 10.1111/j.1728-4465.2010.00231.x 21466110

[46] Anguzu R Sempeera H Sekandi JN. High parity predicts use of long-acting reversible contraceptives in the extended postpartum period among women in rural Uganda. Contracept Reprod Med. (2018) 3:6. 10.1186/s40834-018-0059-8 29760943 PMC5941484

[47] Paul B Ayo AS Ayiga N. Rural-urban contraceptive use in Uganda: evidence from UDHS 2011. J Hum Ecol. (2015) 52:168–82. 10.1080/09709274.2015.11906941

[48] Muhoza DN Ruhara CM. Closing the Poor–Rich Gap in contraceptive use in Rwanda: understanding the underlying mechanisms. Int Perspect Sex Reprod Health. (2019) 45:13–23. 10.1363/45e7519 31592771

[49] Chola L McGee S Tugendhaft A Buchmann E Hofman K. Scaling up family planning to reduce maternal and child mortality: the potential costs and benefits of modern contraceptive use in South Africa. PLoS ONE. (2015) 10:e0130077. 10.1371/journal.pone.0130077 26076482 PMC4468244

[50] Ndugwa RP Kabiru CW Cleland J Beguy D Egondi T Zulu EM. Adolescent problem behavior in Nairobi's informal settlements: applying problem behavior theory in sub-Saharan Africa. J Urban Health. (2011) 88(Suppl. 2):S298–317. 10.1007/s11524-010-9462-4 20499192 PMC3132234

[51] Alhassan N Dodoo FNA. Predictors of primary and secondary sexual abstinence among never-married youth in urban poor Accra, Ghana. Reprod Health. (2020) 17:1–13. 10.1186/s12978-020-0885-4 32085788 PMC7035703

[52] Handa S Palermo T Rosenberg M Pettifor A Halpern CT Thirumurthy H. How does a national poverty programme influence sexual debut among Kenyan adolescents? Glob Public Health. (2017) 12:617–38. 10.1080/17441692.2015.1134617 26853950 PMC4976080

